# Magnetic Resonance Imaging Findings in a Case of Presumed Unilateral Trigeminal Ganglion Aplasia in a 3‐Year‐Old Female French Bulldog

**DOI:** 10.1111/vru.70146

**Published:** 2026-02-14

**Authors:** Jack H. M. Jarvis, Masahiro Murakami, Go Togawa, Jessica E. Linder, Caroline V. Fulkerson

**Affiliations:** ^1^ Department of Veterinary Clinical Sciences, College of Veterinary Medicine Purdue University West Lafayette Indiana USA; ^2^ Department of Small Animal Clinical Sciences, Virginia‐Maryland College of Veterinary Medicine Virginia Polytechnic Institute and State University Blacksburg Virginia USA

**Keywords:** facial anesthesia, keratoconjunctivitis sicca, trigeminal neuropathy

## Abstract

A young female French bulldog with a history of chronic right keratoconjunctivitis sicca (KCS) because since birth was referred for an acute onset of nonambulatory tetraparesis. Right trigeminal nerve sensory deficits, including right corneal anesthesia and absent facial sensation, were identified during examination. Magnetic resonance imaging (MRI) of the neurocranium was performed, revealing severe hypoplasia to the absence of the right trigeminal ganglion, consistent with reports of congenital trigeminal hypoplasia/aplasia in human medicine. This represents a very rare but important differential in young patients with a history of non‐responsive KCS, recurrent or slow healing corneal ulceration, and absent corneal sensation.

## 1 Signalment, History, and Clinical Findings

A 3‐year‐old female French Bulldog was presented for a referral neurological consultation following a 2‐day history of an acute left thoracic limb lameness that progressed to nonambulatory tetraparesis. Additionally, the patient had a long‐standing, nonprogressive history of right keratoconjunctivitis sicca (KCS), reportedly present since birth, and had previously presented to a referral ophthalmology service for recurrent corneal ulceration. At the time of presentation, her KCS was being managed using daily saline flushes and artificial tears. Excluding neurological and ocular findings, on presentation her physical examination was unremarkable. On neurological examination, cervical pain, nonambulatory tetraparesis with moderate to strong motor function in all limbs, decreased withdrawal reflexes with increased muscle tone in the thoracic limbs, and absent to severely delayed paw placement in all limbs were noted, consistent with C6–T2 myelopathy. Concurrent right trigeminal nerve sensory deficits were also noted. Facial sensation, corneal, and palpebral reflexes were assessed bilaterally using a cotton‐tipped applicator, and nasal sensation was assessed using a hemostat. Responses were absent on the right side and normal on the left.

## 2 Imaging, Diagnosis, and Outcomes

**FIGURE 1:**
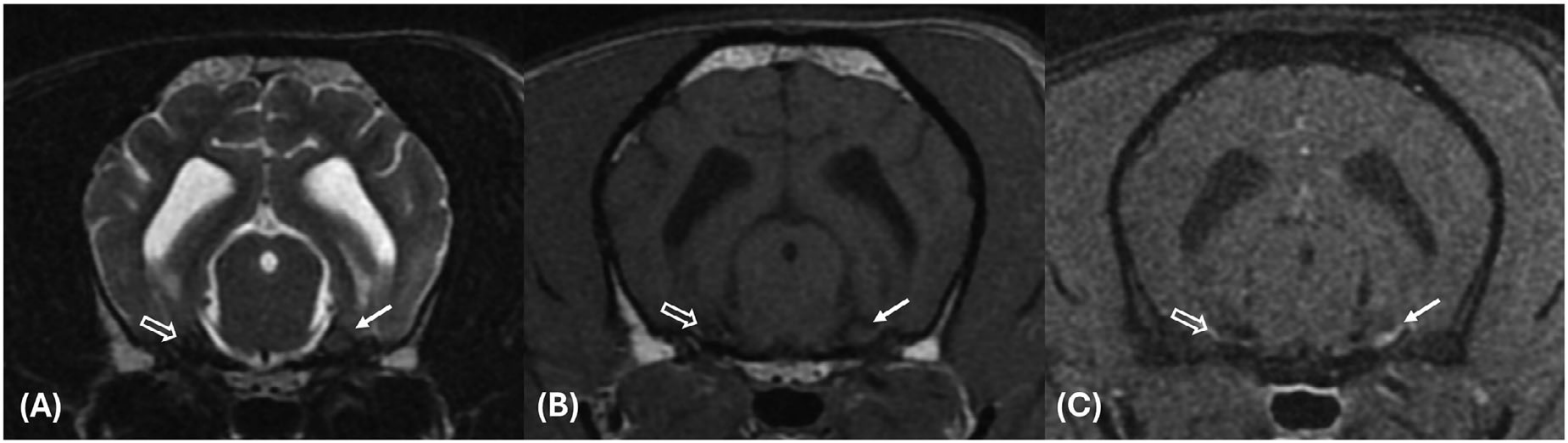
Transverse T2W (A), T1W pre‐contrast (B), and post‐contrast fat‐saturated (C) sequences at the level of the trigeminal nerve (CNV) ganglia and pons showing a normally sized left CNV ganglion (white arrow) and absent right CNV ganglion (open white arrow).

**FIGURE 2:**
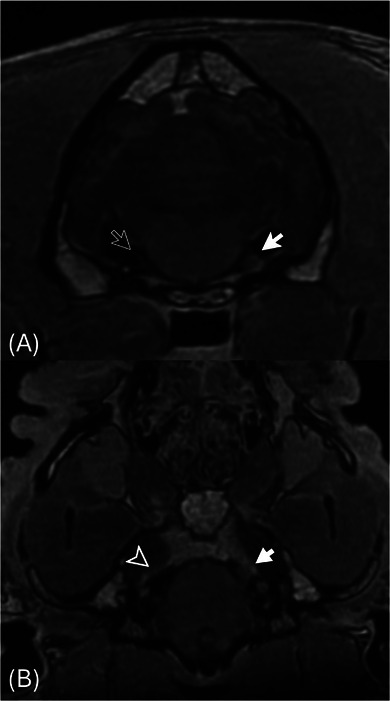
Transverse (A) and oblique dorsal multiplanar reconstruction (B) 3D FSPGR post‐contrast sequences of the trigeminal nerves (CNV) showing the left CNV ganglion (white arrow) and absent right CNV ganglion (open white arrow) in A and course of the left CNV (white arrowhead) and absent right CNV (open white arrowhead) in part (B). Images underwent post‐acquisition denoising using commercial software.

The patient underwent a magnetic resonance imaging (MRI) examination of the brain and cervical spine under general anesthesia with the patient positioned in dorsal recumbency. The study was acquired with a 1.5 T MRI scanner (Signa HDxt, GE Medical Systems, Milwaukee, WI, USA) using an eight‐channel spine coil. Pulse sequences included T1‐weighted (T1W), T2‐weighted (T2W), half Fourier single‐shot turbo spin‐echo (HASTE), and short‐tau inversion recovery images (STIR) of the cervical spine and T1W, T2W, T2*‐weighted gradient echo, T2W fluid‐attenuated inversion recovery (FLAIR), 3D fast spoiled gradient echo (3D FSPGR), and diffusion‐weighted images (DWI) of the brain, with additional cervical and brain post‐contrast T1W fat‐saturated and 3D FSPGR sequences acquired. Brain sequences were acquired with 2.6 mm transverse and dorsal and 2.5 mm sagittal slice thicknesses, except for the 3D FSPGR sequences, which were acquired with 1.2 mm slice thickness. Slice interval gaps of 3.1, 3, 3.3, and 0.6 mm were used for brain axial, sagittal, dorsal, and 3D FSPGR sequences, respectively. Acquisition parameters for the brain pulse sequences were as follows: T1W (TR: 450–762, TE: 9.0–9.6), T2W (TR: 3075–3325, TE: 99.5–103), FLAIR (TR: 8000, TE: 141), T2*‐gradient echo (TR: 384, TE: 5.8, flip angle: 15), 3D FSPGR (TR: 5.42, TE: 1.95, flip angle: 12), and DWI (TR: 10000, TE: 96.3) were obtained with 3 mm slice thickness. Cervical sequences were acquired with 2.5 mm transverse and 2.2 mm sagittal slice thicknesses, with 2.7 and 2.4 mm slice interval gaps for axial and sagittal sequences. Cervical pulse sequences acquisition parameters were T1W (TR: 475 TE: 9.2), T2W (TR: 3660–4500 TE: 83–106), STIR (TR: 3500, TE: 45, IR: 150), and HASTE (TR: 3000, TE: 600). All post‐contrast images were acquired following intra‐venous injection of 0.2 mL/kg gadopentetate dimeglumine (469.01 g/mL, Bayer Healthcare Pharmaceuticals, Wayne, NJ, USA). Images underwent post‐acquisition denoising using commercial denoising deep learning‐based algorithms (HawkAI, HawkCell, Lyon, France) (Figures 1 and 2).

The study revealed a marked reduction in size or absence of the right trigeminal ganglion with a thin, faint T1W and T2W isointense (to grey matter) structure coursing out of the expected location of the origin of the right trigeminal nerve from the pons. No regional compressive tissue was identified. The contralateral trigeminal ganglion was unremarkable in appearance and location, measuring 4.2 mm in diameter. There was no evidence of volume reduction of the masticatory muscles on both sides. The remainder of the brain neuroparenchyma was unremarkable. At the C3–C4 intervertebral disc space, there was evidence of intervertebral disc extrusion causing moderate extradural spinal cord compression with extension into the left C3–C4 intervertebral foramen.

Following the MRI examination, the patient was immediately transported to an operating room and prepared for surgical intervention. A C3–C4 ventral slot technique was performed, resulting in successful spinal cord decompression, and the patient recovered uneventfully from general anesthesia. A day after surgery, an ophthalmology consultation was performed, identifying right‐sided KCS consistent with the previous diagnosis and development of a new left‐sided deep corneal ulceration with a miotic pupil. Subsequently, the left corneal ulceration progressed to a descemetocele, requiring a conjunctival graft, which was performed 3 days after the ventral slot surgery.

## 3 Discussion

This is the first report describing the MRI features of suspected severe trigeminal ganglion hypoplasia to aplasia in a dog. Although neoplastic and inflammatory changes in the trigeminal nerve in canine patients are well reported, based on our systematic review of the veterinary literature, no report of reduced trigeminal ganglion volume and associated trigeminal sensory neuropathy exists [1, 2, 3, 4]. Congenital trigeminal ganglion hypoplasia and aplasia are rarely reported in human medicine, often presenting in pediatric patients with variable clinical signs depending on the branches affected and may occur with other congenital neuroparenchymal abnormalities [5, 6, 7]. In children affected with reduced trigeminal nerve volume, common presenting complaints include corneal ulceration, loss of facial sensation, and absent corneal sensation, also known as corneal anesthesia or hypoesthesia [6, 8, 9] Supporting Information.

The trigeminal nerve is the largest and most complex of the cranial nerves, dividing into the three major branches: The sensory ophthalmic (CNV1) and maxillary (CNV2) nerves and the mixed sensory and motor mandibular nerve (CNV3)10. It arises along the ventrolateral aspect of the pons as a large sensory root and a smaller motor root, coursing into the trigeminal canal. Within the canal, the sensory nerve fibers form the trigeminal ganglion before combining with the motor branches and subsequently dividing into the three major branches distally [10, 11]. These branches exit the skull through three different foramina: The ophthalmic (CNV1) exits via the orbital fissure, the maxillary (CNV2) passes through the round foramen and then exits from the rostral alar foramen, and the mandibular (CNV3) exits from the oval foramen [10, 12]. The MRI features of the normal canine trigeminal nerve are well‐described, correlating closely with anatomical studies [12, 13, 14].

Human pediatric patients with congenital trigeminal nerve hypoplasia often initially present with a history of recurrent corneal injury or infection without evidence of pain [9]. Reported cases most commonly feature just loss of corneal sensory innervation, with a smaller number of cases presenting with concurrent facial anesthesia [9]. Although loss of sensitivity to pain occurs with trigeminal sensory nerve aplasia, the most significant consequence involves the eye and compromise of the ocular protective mechanisms. Branches of CNV1 provide sensation of the cornea, one of the most innervated structures in the body. Additionally, these fibers course alongside postganglionic parasympathetic fibers from the pterygopalatine ganglion and sympathetic fibers from the cranial cervical ganglion to the lacrimal gland [11, 15]. Loss of these nerve fibers results in multiple deleterious effects related to the function of the corneal nerves, which include sensation, blink reflex, and trophic functions [6]. This is further compounded by the interruption of the complex regulation of tear film production, normally tightly controlled from the sensory nerves located on the corneal surface, leading to reduced and imbalanced tear film production from the lacrimal gland, goblet cells, stratified squamous cells, and corneal epithelium [8, 15]. Additionally, corneal innervation is vital for appropriate healing of epithelial defects, reportedly mediated through the release of various healing factors from the corneal nerve endings [6, 8]. The combination of these deficiencies, known as neurotrophic keratitis, results in a spectrum of corneal injury ranging from punctate keratopathy through to corneal rupture and requires lifelong management [6, 8, 9].

In canine patients, KCS is a common ocular disorder, characterized by a decrease in the aqueous component of the tear film and subsequent corneal and conjunctival lesions [16, 17, 18, 19]. KCS often presents in older patients and has numerous potential etiologies, the most common being immune‐mediated destruction of the lacrimal gland, with infectious, toxicological, iatrogenic, traumatic, and congenital causes reported. Congenital alacrima, caused by hypoplasia or agenesis of the lacrimal gland, is a rare cause of KCS with a genetic predisposition for Yorkshire terriers and Bedlington terriers [17, 19]. Similar to this case, this disease often manifests much earlier than the autoimmune variants of KCS, with reported cases diagnosed from 5 months to 4 years of age; however, associated trigeminal nerve defects, including corneal anesthesia, are not reported [19]. Given the broader trigeminal nerve sensory deficits present and MRI findings, reduced lacrimation due to impaired innervation was considered more likely over primary congenital alacrima as a cause for the patient's lifelong history of KCS.

On the basis of our systematic literature review, MRI findings of trigeminal nerve atrophy have not been reported in veterinary literature as a consequence of trigeminal neuropathy. Idiopathic trigeminal neuropathy, neoplasia, and neuritis affecting the trigeminal nerve often cause masticatory muscle atrophy with thickening of the nerve [2, 3]. Ganglioradiculitis has been reported to result in atrophy of the trigeminal nerve; however, this disease is typically associated with additional clinical signs such as ataxia, masticatory muscle wasting, and depressed tendon reflexes, resulting from generalized nonsuppurative inflammation and neuronal loss affecting both ganglia and nerve roots [20]. In the current case, the patient's neurological deficits did not fit the expected pattern for an inflammatory disease. Instead, her clinical findings were best explained by an isolated unilateral trigeminal neuropathy with a concurrent cervical intervertebral disc extrusion, unrelated to the trigeminal deficits. This was further supported by the absence of brain MRI findings consistent with an inflammatory process. Trigeminal nerve atrophy following chronic neurovascular compression is reported in human literature [21]. This was not considered in the current case as no regional compressive tissue was identified, and there was no evidence of trigeminal neuralgia, which is often associated with this disease.

The presence of combined unilateral corneal and facial anesthesia with ipsilateral chronic KCS since an extremely early age in this case report bears striking similarities to the rare findings of congenital trigeminal nerve hypoplasia reported in people. When paired with the MRI findings of absent to severely reduced volume of the right trigeminal ganglion, a presumptive diagnosis of unilateral congenital trigeminal ganglion aplasia was made.

There are several limitations to this case report, the foremost of which is the presumptive antemortem diagnosis of trigeminal ganglion aplasia without histopathological confirmation. With the patient's age at presentation, an early life insult to the right trigeminal ganglion cannot be excluded. Additionally, due to limited follow‐up, no sequential MR imaging studies were performed to assess for changes over time. Although the use of different imaging acquisition devices (e.g., a higher Tesla MR system, dedicated higher channel brain coil) may have improved imaging quality, this was considered unlikely to change the final presumptive diagnosis in this case.

## 4 Conclusion

This case presents the first report of presumed unilateral trigeminal ganglion aplasia in a young female French Bulldog resulting in ipsilateral corneal and facial anesthesia and associated recurrent KCS. Given the similarities in presentation to human cases and the rarer congenital forms of KCS, it is important to consider this differential in young patients with a history of treatment‐refractory KCS, recurrent or slow healing corneal ulceration, and absent corneal sensation.

## Author Contributions

Conception and design: Jack H. M. Jarvis. Acquisition of data: Jack H. M. Jarvis, Go Togawa, and Jessica E. Linder. Analysis and interpretation of data: Jack H. M. Jarvis, Caroline V. Fulkerson, Masahiro Murakami, Go Togawa, and Jessica E. Linder. Drafting the article: Jack H. M. Jarvis. Revising article for intellectual content: Jack H. M. Jarvis, Caroline V. Fulkerson, Masahiro Murakami, Go Togawa, and Jessica E. Linder. Final approval of the completed article: Jack H. M. Jarvis, Caroline V. Fulkerson, Masahiro Murakami, Go Togawa, and Jessica E. Linder. Agreement to be accountable for all aspects of the work in ensuring that questions related to the accuracy or integrity of any part of the work are appropriately investigated and resolved: Jack H. M. Jarvis, Caroline V. Fulkerson, Masahiro Murakami, Go Togawa, and Jessica E. Linder.

## Disclosure

This study has not been previously presented or published in an abstract.

## Conflicts of Interest

The authors declare no conflicts of interest.

## Supporting information




**Video S1**: Transverse 3D FSPGE post‐contrast sequences through the level of the trigeminal ganglion, demonstrating the absence of parenchyma in the expected location of the right CNV ganglion compared to the left. Images underwent post‐acquisition denoising using commercial software.

## Data Availability

The data are available to readers from the corresponding author upon reasonable request.
